# Impact of lymphopenia and hypogammaglobulinemia on outcomes in neutropenic patients with hematological malignancies

**DOI:** 10.1007/s12185-025-04120-y

**Published:** 2025-12-05

**Authors:** Andry Van de Louw, Lauren King, Myles Nickolich

**Affiliations:** 1 Division of Pulmonary and Critical Care Medicine, Penn State Health Hershey Medical Center, Hershey, PA 17033 USA; 2 Division of Hematology and Oncology, Penn State Health Hershey Medical Center, Hershey, PA 17033 USA

**Keywords:** Hypogammaglobulinemia, Lymphopenia, Neutropenia, Lymphoma, Leukemia

## Abstract

Neutropenic patients with hematological malignancies are at high risk for infectious complications. Whether associated lymphopenia or hypogammaglobulinemia further increase this risk and affect outcome remains unclear. This retrospective single-center study included 321 patients hospitalized with lymphoma or leukemia and severe neutropenia whose serum immunoglobulin levels were measured. Overall, 60% of patients had isolated lymphopenia, 9% isolated hypogammaglobulinemia, 24% both, and 7% none. There was no correlation between absolute lymphocyte count and IgG. Severe lymphopenia was not associated with infection or mortality. Hypogammaglobulinemia was more prevalent in lymphoid leukemias and lymphomas. A multivariate logistic regression analysis adjusting for age, underlying hematological malignancy, absolute neutrophil and lymphocyte counts, and duration of neutropenia and lymphopenia showed that hypogammaglobulinemia was associated with infection (OR 1.85, 95% CI 1.07–3.26, *p* = 0.03), pneumonia (OR 2.04, 95% CI 1.13–3.72, *p* = 0.02), and sepsis or septic shock (OR 2.43, 95% CI 1.26–4.72, *p* = 0.01). This indicates a need for further investigation into the role of immunoglobulin replacement therapy in this setting.

**Supplementary Information:**

The online version contains supplementary material available at 10.1007/s12185-025-04120-y.

## Introduction

Infections are a frequent complication of hematological malignancies, associated with significant morbidity, mortality, and potential delays in treatment.

Neutropenia is a major risk factor for infections: the quantitative relationship between the severity of neutropenia, the risk of developing infection and its associated mortality has long been recognized [1]. Febrile neutropenia carries a mortality of around 10% overall [2] and as high as 50% if septic shock is present [3].

Other immune deficiencies, such as quantitative or qualitative defect of lymphocyte function, may also increase the risk of infection and death. Lymphopenia has been associated with increased risk of infection and mortality in different populations, including patients with hematological malignancies [4, 5]. Hypogammaglobulinemia has also been associated with increased risk of infections mostly in patients with B-cell-derived malignancies such as chronic lymphocytic leukemia (CLL), multiple myeloma (MM), and non-Hodgkin lymphoma (NHL) [6]. Monitoring of serum immunoglobulin G (IgG) levels in patients with hematological malignancies undergoing anti-cancer therapy has been recommended by some experts [7].

Studies have shown that lymphopenia was a risk factor for febrile neutropenia after chemotherapy in patients with diverse cancers [8], but the prevalence of lymphopenia and hypogammaglobulinemia and how they interplay with severe neutropenia to affect infection and death rates in hematological malignancies has been poorly investigated.

The objective of this study was to assess the association between IgG levels, blood absolute lymphocyte count (ALC) and outcome (neutropenic fever, infectious complications, mortality) in neutropenic patients with hematological malignancies.

## Methods

The study was approved by the Pennsylvania University IRB (study number 19227) and informed consent was waived due to the retrospective nature of data collection. All adult (≥ 18 years) patients admitted to Penn State Health Hershey Medical Center between 2005 and 2020 with a diagnosis of hematological malignancy (ICD-9 200–208.99 or ICD-10 C81–C96) and who had a blood absolute neutrophil count (ANC) ≤ 500/mm^3^ during admission were screened. Patients with acute or chronic myeloid or lymphoid leukemia, Hodgkin and non-Hodgkin lymphoma who had serum immunoglobulins (Ig) levels measured during admission were included; patients with Ig measured after Ig replacement therapy (IgRT) were excluded. For patients with multiple eligible admissions, only the admission with the lowest IgG level was included as a surrogate for the most severe degree of humoral immunodeficiency.

The following variables were collected: age, gender, underlying hematological malignancy and history of hematopoietic stem cell transplant (HSCT), ICD-9 or ICD-10 diagnoses for infections, temperature throughout admission, ANC and ALC throughout the admission, serum IgG, IgA and IgM levels, administration of IgRT, hospital length of stay and mortality. Chemotherapy and other immunosuppressants with potential effect on blood cell counts (targeted therapy, immunotherapy, antirejection drugs) received during admission were also collected. Lymphopenia was defined as ALC ≤ 500/mm^3^ and hypogammaglobulinemia as serum IgG ≤ 500 mg/dL. The need for vasopressor therapy, invasive mechanical ventilation, and renal replacement therapy was collected. Neutropenic fever was defined as a single temperature measurement of ≥ 38.3 °C (101°F) or a temperature of ≥ 38.0 °C (100.4 °F) sustained over a 1-h period, along with an ANC ≤ 500/mm^3^ [9]. For the comparison of outcomes between hematological malignancies, 3 groups were considered (lymphomas, lymphoid leukemias and myeloid leukemias). Infections were defined as bacterial, viral or fungal (including candidosis and aspergillosis) and a group of pneumonias was added due to the frequency of ICD codes for pneumonia not otherwise specified. In accordance with our institutional protocols, neutropenic patients with acute myeloid leukemia and HSCT recipients received antimicrobial prophylaxis with antivirals (acyclovir or valacyclovir), antibiotics (ciprofloxacin or levofloxacin) and antifungals (posaconazole, voriconazole, fluconazole or isavuconazole).

### Statistical analysis

Continuous and categorical variables were reported as median (interquartile range) and number (percentage) respectively and compared between groups using Wilcoxon rank-sum test of Fisher exact test as appropriate. As ALC and serum IgG were not normally distributed, a Spearman rank correlation test was used to assess the correlation between these two variables. Multivariate logistic regression analyses were used to analyze the association between hypogammaglobulinemia and infectious complications, pneumonias and sepsis or septic shock; linearity assumption, effect of outliers and multicollinearity were carefully checked.

As including only patients who had IgG measured was a potential source of selection bias, we performed sensitivity analyses to take into account the odds of having IgG measured. Inverse probability weighting (IPW) logistic regression was used by first modeling the odds of IgG measurements for all admissions of neutropenic patients with qualifying hematological malignancies. This first model included the underlying hematological malignancy, HSCT, ALC, ANC, and documented infection as covariates. It allowed us to create weights for each admission based on the odds of having IgG measured. The sensitivity analysis consisted of the same logistic regression as our main model but performed on weighted observations of patients with IgG available.

A p value threshold of < 0.05 was considered significant, and all tests were two-sided. Statistical analyses were performed using R package (version 2022.07.01).

## Results

**Fig. 1:**
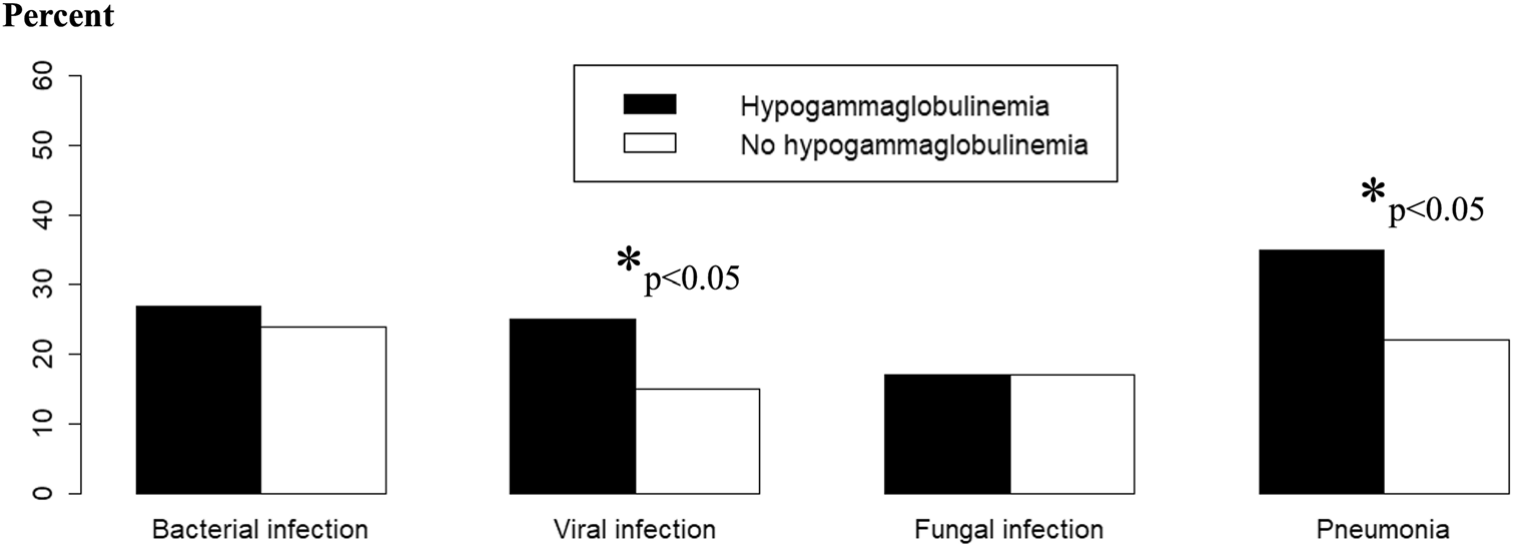
Proportion of patients who developed infections (bacterial, viral, fungal, pneumonia not otherwise specified) according to the presence of hypogammaglobulinemia. Patients with hypogammaglobulinemia more frequently developed viral infections and pneumonias

During the study period, there were 6176 admissions of 2577 neutropenic patients with hematological malignancy (multiple myeloma excluded). IgG was obtained for 406 of these admissions. Table 1S provides a comparison of admissions with and without IgG measurements: admissions with IgG available involved younger patients, more frequently with lymphoid leukemia and HSCT, with more profound lymphopenia and neutropenia, more documented infections and increased severity, as reflected by higher requirements for vital organ support and higher mortality. After excluding patients without leukemia or lymphoma and patients who received IVIG prior to IgG measurement and selecting unique admissions with lowest IgG levels, our final population included 321 patients (study flowchart in Fig. 1S).

### Association between lymphopenia, hypogammaglobulinemia, and outcome

Patients’ characteristics are described in Table 1. Patients were admitted for myeloid leukemia (46%), lymphoid leukemia (28%), and lymphoma (25%); 64% and 57% of patients received inpatient chemotherapy and other immunosuppressants, respectively. Overall, 60% of patients had isolated lymphopenia, 9% isolated hypogammaglobulinemia, 24% both, and 7% none. There was no correlation between ALC and IgG (correlation coefficient 0.018, *p* = 0.74) even when only including patients with myeloid leukemia (correlation coefficient 0.20, *p* = 0.02). For instance, 40% of patients with lymphopenia (ALC ≤ 500/mm^3^) had normal IgG levels (> 700 mg/dL) and 66% of patients with ALC > 1000/mm^3^ had IgG levels < 500 mg/dL. Among patients with hypogammaglobulinemia, 67% and 72% also had low levels of IgM and IgA respectively.

**Table 1:** Characteristics of the study population

	All patients (*n* = 321)
Gender, *n* (%)	
Female	139 (43.3%)
Male	182 (56.7%)
Age (years)	53 (40–65)
Hematological malignancy, *n* (%)	
Lymphoid leukemia	89 (27.7%)
Lymphoma	80 (24.9%)
Myeloid leukemia	147 (45.8%)
Other	5 (1.6%)
Hematopoietic stem cell transplant, *n* (%)	144 (44.9%)
Serum IgG (mg/dL)	631 (432–897)
Hypogammaglobulinemia, *n* (%)	106 (33.0%)
Lymphocyte count (× 10^9^/L)	0.10 (0.01–0.30)
Lymphopenia, *n* (%)	267 (83.2%)
Duration of lymphopenia (days)	13 (5–19)
Neutrophil count (× 10^9^/L)	0.20 (0.04–0.39)
Duration of neutropenia (days)	6 (3–10)
Administration of IVIG, *n* (%)	33 (10.3%)
Inpatient chemotherapy, *n* (%)	204 (64.0%)
Treatment with other immunosuppressants, *n* (%)	184 (57.3%)
Infectious complications, *n* (%)	198 (61.7%)
Neutropenic fever, *n* (%)	130 (40.5%)
Sepsis or septic shock, *n* (%)	60 (18.7%)
Vasopressors, *n* (%)	41 (12.8%)
Invasive mechanical ventilation, *n* (%)	36 (11.2%)
Renal replacement therapy, *n* (%)	9 (2.8%)
Length of hospital stay (days)	22 (13–27)
Hospital mortality, *n* (%)	29 (9.0%)

Patients with lymphopenia had higher IgG levels (645 [454–902] versus 471 [303–858] mg/dL, *p* = 0.02), higher ANC (0.24 [0.10–0.40] versus 0.03 [0.00–0.13] × 10^9^/L, *p* < 0.001) and lower prevalence of hypogammaglobulinemia (28.5% versus 55.6%, *p* < 0.001) and infections (58.4% versus 77.8%, *p* = 0.008) than their counterparts. No significant difference in requirements for vasopressors or invasive mechanical ventilation and hospital mortality was observed between the two groups.

Patients with hypogammaglobulinemia had more frequently lymphoid leukemia or lymphoma, and less frequently myeloid leukemia (Table 2). Their lowest ALC, lowest ANC, and prevalence of severe lymphopenia (ALC < 0.2 × 10^9^/L) was not different from patients without hypogammaglobulinemia. They received IVIG more frequently and developed more infectious complications, including sepsis or septic shock. Specifically, viral infections and pneumonias were more frequent in that group (Fig. 1). Patients with hypogammaglobulinemia required more frequently invasive mechanical ventilation or vasopressor therapy and their hospital mortality was higher than patients without hypogammaglobulinemia (Table 2).

**Table 2:** Comparison of patients’ characteristics according to the presence of hypogammaglobulinemia

	No hypogammaglobulinemia (*N* = 215)	Hypogammaglobulinemia (*N* = 106)	*p* value
Gender, *n* (%)			0.487
Female	96 (44.7%)	43 (40.6%)	
Male	119 (55.3%)	63 (59.4%)	
Age (years)	53 (40–65)	56 (40–67)	0.314
Hematological malignancy, *n* (%)			
Lymphoid leukemia	44 (20.5%)	45 (42.5%)	
Lymphoma	44 (20.4%)	36 (34.0%)	
Myeloid leukemia	123 (57.2%)	24 (22.6%)	
Other	4 (1.9%)	1 (0.9%)	
Hematopoietic stem cell transplant, *n* (%)	106 (49.3%)	38 (35.8%)	0.02
Serum IgG (mg/dL)	769 (629–1049)	378 (296–429)	< 0.001
Serum IgA^a^ (mg/dL)	131 (74–227)	40 (25–71)	< 0.001
Serum IgM^b^ (mg/dL)	50 (30–107)	26 (25–47)	< 0.001
Lymphocyte count (× 10^9^/L)	0.10 (0.01–0.30)	0.10 (0.00–0.53)	0.476
Lymphopenia, *n* (%)	191 (88.8%)	76 (71.7%)	< 0.001
Duration of lymphopenia (days)	13 (6–18)	12 (0–21)	0.30
ALC < 0.2 × 10^9^/L, *n* (%)	141 (65.6%)	68 (64.2%)	0.80
Neutrophil count (× 10^9^/L)	0.20 (0.06–0.40)	0.20 (0.02–0.33)	0.337
Duration of neutropenia (days)	6 (3–11)	6 (3–8)	0.17
Administration of IVIG, *n* (%)	11 (5.1%)	22 (20.8%)	< 0.001
Infectious complications, *n* (%)	122 (56.7%)	76 (71.7%)	0.010
Neutropenic fever, *n* (%)	131 (60.9%)	60 (56.6%)	0.458
Sepsis or septic shock, *n* (%)	30 (14.0%)	30 (28.3%)	0.002
Vasopressors, *n* (%)	18 (8.4%)	23 (21.7%)	< 0.001
Invasive mechanical ventilation, *n* (%)	13 (6.0%)	23 (21.7%)	< 0.001
Renal replacement therapy, *n* (%)	6 (2.8%)	3 (2.8%)	0.984
Length of hospital stay (days)	22 (16–27)	22 (10–30)	0.52
Hospital mortality, *n* (%)	14 (6.5%)	15 (14.2%)	0.025

^a^*n* = 120; ^b^*n* = 114

A comparison of patients who did and did not develop infectious complications is provided in Table 3. Hypogammaglobulinemia was more prevalent in patients with infections despite higher ALC and a lower prevalence of lymphopenia; ANC was not different between the two groups. Patients with infections received more frequently IVIG, required more vasopressor and invasive mechanical ventilation, and had higher hospital mortality. In a multivariate logistic regression analysis adjusting for age, underlying hematological malignancy, ALC, and ANC, duration of neutropenia and lymphopenia, factors associated with infections were age, neutropenia duration and hypogammaglobulinemia (OR 1.85, 95% CI 1.07–3.26, *p* = 0.03) (Table 4); hypogammaglobulinemia was more specifically associated with pneumonias (OR 2.04, 95% CI 1.13–3.72, *p* = 0.02). Hypogammaglobulinemia was also associated with sepsis or septic shock after adjusting for the same covariates (OR 2.43, 95% CI 1.26–4.72, *p* = 0.008). HSCT was not included in the models due to multicollinearity.

**Table 3:** Comparison of patients based on the occurrence of infectious complications

	No infection (*n* = 123)	Infections (*n* = 198)	*p* value
Gender, *n* (%)			0.323
Female	49 (39.8%)	90 (45.5%)	
Male	74 (60.2%)	108 (54.5%)	
Age (years)	51 (38–61)	56 (42–68)	0.020
Hematological malignancy, *n* (%)			0.114
Lymphoid leukemia	31 (25.2%)	58 (29.3%)	
Lymphoma	24 (19.5%)	56 (28.3%)	
Myeloid leukemia	65 (52.8%)	82 (41.4%)	
Other	3 (2.4%)	2 (1.0%)	
Hematopoietic stem cell transplant, *n* (%)	86 (69.9%)	58 (29.3%)	< 0.001
Serum IgG (mg/dL)	631 (509–853)	632 (399–925)	0.264
Hypogammaglobulinemia, *n* (%)	30 (24.4%)	76 (38.4%)	0.010
Lymphocyte count (× 10^9^/L)	0.05 (0.00–0.16)	0.14 (0.02–0.42)	< 0.001
Lymphopenia, *n* (%)	111 (90.2%)	156 (78.8%)	0.008
Duration of lymphopenia (days)	15 (7–18)	12 (3–20)	0.28
Neutrophil count (× 10^9^/L)	0.22 (0.08–0.39)	0.20 (0.02–0.39)	0.207
Duration of neutropenia (days)	6 (3–9)	6 (3–13)	0.14
Administration of IVIG, *n* (%)	2 (1.6%)	31 (15.7%)	< 0.001
Vasopressors, *n* (%)	2 (1.6%)	39 (19.7%)	< 0.001
Invasive mechanical ventilation, *n* (%)	3 (2.4%)	33 (16.7%)	< 0.001
Renal replacement therapy, *n* (%)	1 (0.8%)	8 (4.0%)	0.089
Length of hospital stay (days)	22 (17–24)	23 (12–34)	0.077
Hospital mortality, *n* (%)	1 (0.8%)	28 (14.1%)	< 0.001

**Table 4:** Multivariate logistic regression describing the variables associated with the occurrence of infections

Covariate	OR (95% CI)	*p*
Age (years)	1.02 (1.00–1.03)	0.02
Underlying malignancy (ref: lymphoid leukemia) * Lymphoma* * Myeloid leukemia*	1.15 (0.57–2.32) 0.64 (0.34–1.21)	0.70 0.20
Absolute neutrophil count	1.16 (0.99–1.50)	0.13
Duration of neutropenia	1.09 (1.04–1.14)	< 0.001
Absolute lymphocyte count	1.00 (0.99–1.01)	0.70
Duration of lymphopenia	1.00 (0.97–1.02)	> 0.90
Hypogammaglobulinemia	1.85 (1.07–3.26)	0.03

In the sensitivity analysis (same logistic regression model performed on weighted observations based on the odds of having IgG measured), hypogammaglobulinemia remained associated with infections (OR 1.83, 95% CI 1.65–2.03, *p* < 0.001), pneumonias (OR 2.26, 95% CI 2.01–2.54, *p* < 0.001) and sepsis or septic shock (OR 2.26, 95% CI 2.00–2.57, *p* < 0.001).

### Comparison of lymphomas, lymphoid, and myeloid leukemias

**Fig. 2:**
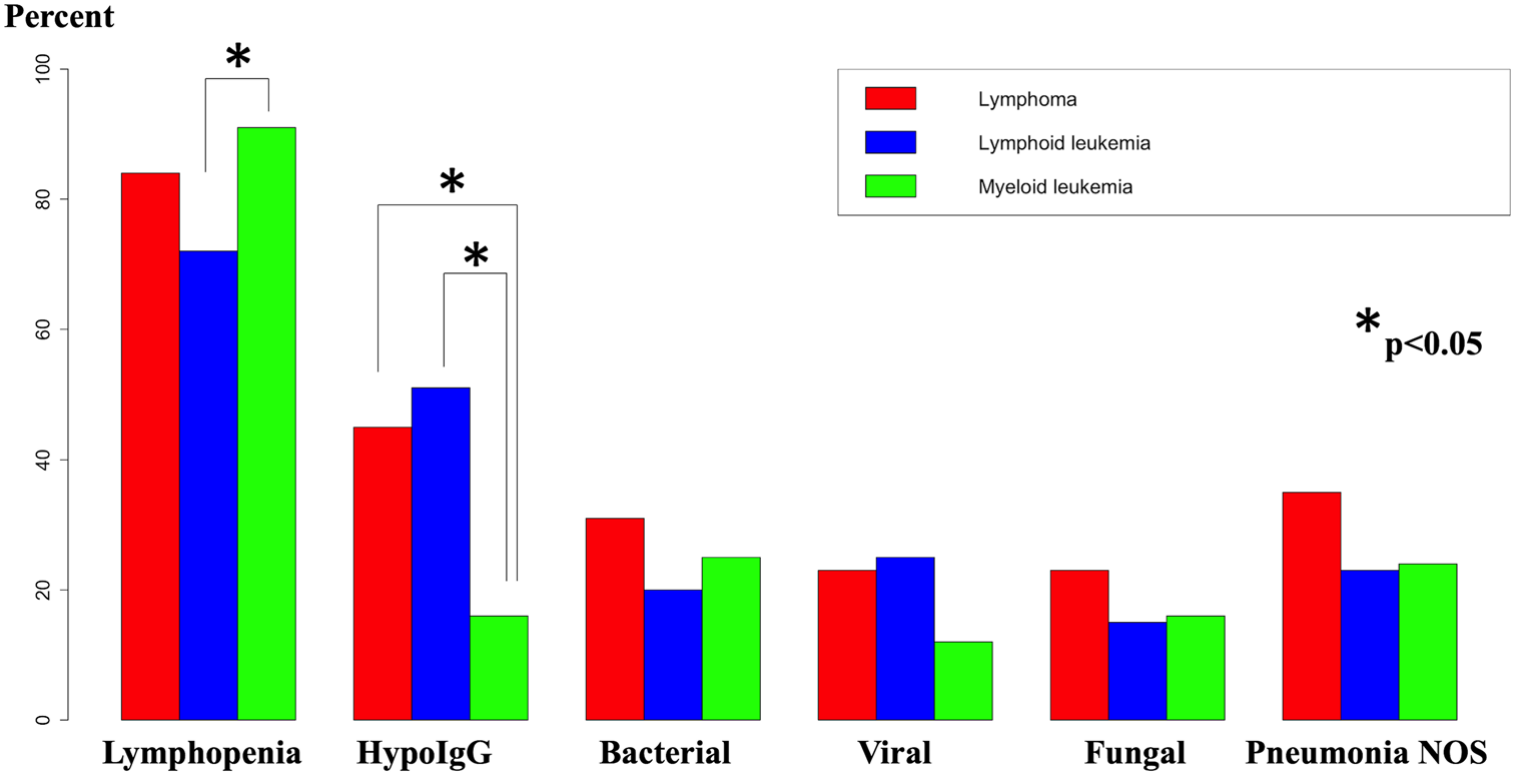
Proportion of patients in the three groups of hematological malignancies (lymphoma, lymphoid leukemia, myeloid leukemia) who had lymphopenia, hypogammaglobulinemia and developed bacterial, viral, fungal infections and pneumonias not otherwise specified. Lymphopenia was significantly more frequent in myeloid than lymphoid leukemias and hypogammaglobulinemia was more frequent in lymphoid leukemia and lymphoma as compared to myeloid leukemias

When comparing patients based on underlying malignancy, lymphopenia was more prevalent in myeloid leukemias than lymphoid leukemias and hypogammaglobulinemia was less prevalent in myeloid leukemia than either lymphoid leukemias or lymphomas (Fig. 2). There was a significant difference in viral infections between the three groups, but pairwise comparisons did not reach significance (*p* = 0.06 for lymphoid versus myeloid leukemias).

### Infections and IVIG administration

Among the 106 patients with hypogammaglobulinemia, 76 (72%) developed infections. The most frequent infections were: pneumonia (*n* = 30), candidiasis (*n* = 9), *Clostridioides difficile* colitis (*n* = 9), HSV infections (*n* = 9), invasive pulmonary aspergillosis (*n* = 8), upper respiratory tract infection (*n* = 8), *Cytomegalovirus* infection (*n* = 7) and urinary tract infection (*n* = 7). Twenty-four patients developed sepsis and 6 septic shock. Twenty-two patients received IVIG during admission: as compared to hypogammaglobulinemic patients who did not receive IVIG, they had lower IgG levels and more infectious complications (Table 5). However, for the 21 of these 22 patients who developed infections, IVIG were always administered after infections were diagnosed. There was no significant difference in outcomes (neutropenic fever, vital organ support and hospital mortality) between the two groups.

**Table 5:** Comparison of patients with hypogammaglobulinemia who received and did not receive IVIG

	No IVIG (*n* = 84)	IVIG (*n* = 22)	*p* value
Gender, *n* (%)			0.60
Female	33 (39.3%)	10 (45.5%)	
Male	51 (60.7%)	12 (54.5%)	
Age (years)	56 (41–67)	55 (37–64)	0.74
Hematological malignancy, *n* (%)			0.61
Lymphoid leukemia	37 (44%)	8 (36%)	
Lymphoma	26 (31%)	10 (50%)	
Myeloid leukemia	20 (24%)	4 (18%)	
Other	1 (1%)	0 (0%)	
Hematopoietic stem cell transplant, *n* (%)	36 (43%)	2 (9%)	0.003
Serum IgG (mg/dL)	384 (320–432)	292 (220–396)	0.03
Lymphocyte count (× 10^9^/L)	0.10 (0.00–0.59)	0.12 (0.03–0.23)	0.91
Lymphopenia, *n* (%)	59 (70%)	17 (77%)	0.51
Duration of lymphopenia (days)	12 (0–19)	13 (4–30)	0.29
Neutrophil count (× 10^9^/L)	0.20 (0.01–0.34)	0.24 (0.14–0.31)	0.22
Duration of neutropenia (days)	6 (3–8)	6 (2–8)	0.53
Infectious complications, *n* (%)	55 (66%)	21 (96%)	0.005
Neutropenic fever, *n* (%)	47 (56%)	13 (59%)	0.79
Vasopressors, *n* (%)	17 (20%)	6 (27%)	0.48
Invasive mechanical ventilation, *n* (%)	17 (20%)	6 (27%)	0.48
Renal replacement therapy, *n* (%)	2 (2%)	1 (5%)	0.59
Hospital mortality, *n* (%)	14 (17%)	1 (5%)	0.15

## Discussion

The main findings of this study were that in a population of hospitalized, neutropenic patients with hematological malignancies (multiple myeloma excluded), associated lymphopenia and hypogammaglobulinemia were present in about 80% and 30% of patients, respectively. Whereas lymphopenia was not associated with outcomes, patients with hypogammaglobulinemia developed more infections, including sepsis, required more vital organ support, and had higher mortality. Hypogammaglobulinemia was an independent risk factor for infections and sepsis.

A recent literature review highlighted the underreporting of rates of lymphopenia and even more so hypogammaglobulinemia in patients with CLL, MM, and NHL [10]. Their impact on infectious risk has been investigated in specific malignancies, but never under the angle of additional risk factor in patients already immunocompromised by their neutropenia. To our knowledge, the present study is the first to report the rates of lymphopenia and hypogammaglobulinemia in neutropenic patients with hematological malignancies and to show that, in this population already at high risk of infection, additional hypogammaglobulinemia (but not lymphopenia) further increased the risk of infection and sepsis.

Lymphopenia has been associated with increased risks of severe infection and mortality in various populations [11] including patients with immunodeficiencies/hematological diseases [4]. ALC < 1.0 × 10^9^/L was observed in about 25% of patients with diffuse large B-cell lymphoma before treatment with an independent impact on prognosis [12]. Lymphopenia and depletion of memory B-cell compartment have also been documented in non-cancer patients with sepsis and associated with mortality [13, 14]. Limited data exist on the additional impact of lymphopenia in patients already immunocompromised due to neutropenia: a study in 210 critically ill neutropenic patients observed that severe lymphopenia (ALC < 0.5 × 10^9^/L) was present in 65% of patients but neither ALC nor kinetics of blood lymphocyte counts seemed associated with mortality [15]. The present study confirmed that severe lymphopenia was extremely prevalent in neutropenic patients with lymphomas and leukemias, even outside of the ICU, but did not seem associated with an increased infectious risk or mortality.

There was no strong correlation in our study between ALC and serum IgG in agreement with other reports [16], so that patients with profound lymphopenia could still have maintained IgG levels, perhaps accounting for the lack of strong association between ALC and outcome. During HIV infection, a weak correlation has been reported between serum Ig levels and number of Ig-secreting cells as well as an inverse correlation between Ig-secreting B cells and other lymphocytes like CD4 cells [17], so that it would be oversimplistic to view Ig levels as a direct function of total ALC.

Contrary to lymphopenia, hypogammaglobulinemia in our population was independently associated with increased risk of infection and sepsis. Hypogammaglobulinemic patients also required more vital organ support in the ICU and had higher hospital mortality in unadjusted analyses but assessing the potential impact of hypogammaglobulinemia on mortality would require robust prospective studies. Hypogammaglobulinemia has been similarly associated with infection and outcome in solid organ transplant recipients [18], after chimeric antigen receptor T-cell therapy [19] or rituximab administration [20] or in patients with CLL [21] or lymphoma [22]. However, its impact in hospitalized patients who already had a severe defect in neutrophil function has not been investigated, and we confirmed that even in this population, low IgG levels independently affect outcomes.

We observed that hypogammaglobulinemia was significantly more frequent in lymphomas and lymphoid leukemia as opposed to myeloid leukemia. This is consistent with the literature where studies on secondary immune deficiency have mostly focused on chronic lymphocytic leukemia, non-Hodgkin lymphoma, and multiple myeloma [6]. However, hypogammaglobulinemia has been reported in patients with chronic myeloid leukemia treated with imatinib [16] or in patients after hematopoietic stem cell transplant [23], which likely account for the proportion of patients with myeloid leukemia found to have hypogammaglobulinemia in the present study. We decided to exclude patients with multiple myeloma because some experts do not recommend routine IgG measurements in this population, due to the paraprotein secreted interfering with functional Ig measurements [7].

The patients who received IVIG in our study had lower IgG levels and more infections than patients who did not receive IVIG. However, all infected patients who received IVIG did so after infections were diagnosed, so that IVIG were presumably used as a way to facilitate the resolution of infections in our population, rather than to prevent them. In agreement with previous studies [18], respiratory tract infections, CMV, aspergillosis, and other fungal infections were the most frequent infections in hypogammaglobulinemic patients. Although hospital mortality appeared lower in patients who received IVIG (5% versus 17%), the difference did not reach statistical significance due to the small sample size. The analysis was not adjusted for potential confounders, so that no causal inference can be made.

Our study has several limitations. Its single-center design limits its generalizability. By design, only patients whose Ig levels were measured were included, which could represent a selection bias. Indeed, infections were more frequent during admissions with Ig measurements available, possibly because Ig were measured preferentially in patients with suspected or confirmed infections. To mitigate this potential bias, we used IPW logistic regression to account for the odds of having Ig obtained and still confirmed an association between hypogammaglobulinemia and infectious complications, pneumonia or sepsis. However, our findings may not be generalizable to unselected neutropenic patients with hematological malignancies and should be confirmed in more standardized studies. IVIG administration was at the discretion of attending physicians, which makes its impact difficult to interpret. The reliability of infection diagnoses relied on the accuracy of ICD codes, which may have been underreported. Finally, although our study showed that hypogammaglobulinemia, unlike lymphopenia, increased the risk of infection and sepsis in neutropenic patients, the size of our population did not allow us to explore the complex interplay between the severity and duration of neutropenia, lymphopenia, and hypogammaglobulinemia and whether additive or multiplicative interactions may exist between these risk factors.

In summary, in neutropenic inpatients with hematological malignancies, we observed that lymphopenia and hypogammaglobulinemia were very common, especially in patients with lymphomas or lymphoid leukemias. While lymphopenia was not associated with outcomes, hypogammaglobulinemia, when tested for, was an independent risk factor for infections and sepsis. These findings suggest that routine monitoring of IgG levels and the proactive use of targeted immunoglobulin replacement therapy may reduce infection-related morbidity and mortality in neutropenic patients with lymphoid malignancies. Prospective, controlled studies are warranted to validate the clinical benefit of this approach.

## Supplementary Information

Below is the link to the electronic supplementary material.

Supplementary Material 1

Supplementary Material 2

## Data Availability

The data supporting the findings of this study are available from the corresponding author upon reasonable request.
