# Supplementary material for: Impact of lymphopenia and hypogammaglobulinemia on outcomes in neutropenic patients with hematological malignancies
Source: Int J Hematol. 2025 Dec 5;123(3):356–63. doi: 10.1007/s12185-025-04120-y (PMC12967544; doi:10.1007/s12185-025-04120-y)
Supplement: Supplementary file 1 — Supplementary Material 1 [file 12185_2025_4120_MOESM1_ESM.docx]

**Table 1S: comparison of admissions with and without IgG measurement obtained.**

|  | **No IgG measurement (n=5770)** | **IgG measured (n=406)** | **p value** |
| --- | --- | --- | --- |
| **Gender, n (%)** |  |  | 0.954 |
| Female | 2396 (41.5%) | 168 (41.4%) |  |
| Male | 3374 (58.5%) | 238 (58.6%) |  |
| **Age (years)** | 59 (45-68) | 52 (35-65) | < 0.001 |
| **Hematological malignancy, n (%)** |  |  | < 0.001 |
| Lymphoid leukemia | 921 (16.0%) | 114 (28.1%) |  |
| Lymphoma | 1563 (27.1%) | 103 (25.4%) |  |
| Myeloid leukemia | 3231 (56.0%) | 183 (45.1%) |  |
| Other | 55 (1%) | 6 (1.5%) |  |
| **Hematopoietic stem cell transplant,**  **n (%)** | 609 (10.6%) | 164 (40.4%) | < 0.001 |
| **Lymphocyte count (x10^9^/L)** | 0.03 (0.00-0.40) | 0.00 (0.00-0.27) | < 0.001 |
| **Neutrophil count (x10^9^/L)** | 0.00 (0.00-0.22) | 0.00 (0.00-0.11) | < 0.001 |
| **Lymphopenia (ALC < 0.5x10^9^/L)** | 4614 (80.0%) | 340 (83.7%) | 0.065 |
| **Profound neutropenia**  **(ANC < 0.1x10^9^/L)** | 3703 (64.2%) | 297 (73.2%) | < 0.001 |
| **Infectious complications, n (%)** | 2640 (45.8%) | 261 (64.3%) | < 0.001 |
| **Sepsis or septic shock, n (%)** | 315 (11.9%) | 44 (16.9%) | 0.021 |
| **Invasive mechanical ventilation, n (%)** | 403 (7.0%) | 54 (13.3%) | < 0.001 |
| **Renal replacement therapy, n (%)** | 82 (1.4%) | 15 (3.7%) | < 0.001 |
| **Vasopressors, n (%)** | 294 (5.1%) | 63 (15.5%) | < 0.001 |
| **Hospital mortality, n (%)** | 424 (7.3%) | 46 (11.3%) | 0.003 |
