# Supplementary figures and images for: Impact of lymphopenia and hypogammaglobulinemia on outcomes in neutropenic patients with hematological malignancies
Source: Int J Hematol. 2025 Dec 5;123(3):356–63. doi: 10.1007/s12185-025-04120-y (PMC12967544; doi:10.1007/s12185-025-04120-y)

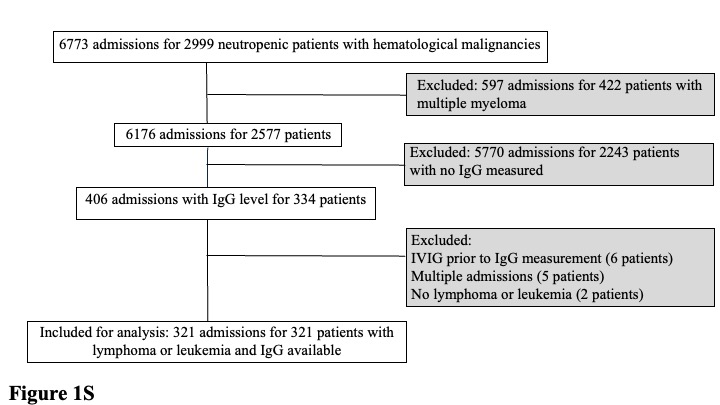

Supplement: Supplementary file 2 — Supplementary Material 2 [file 12185_2025_4120_MOESM2_ESM.jpeg]
